# Spermatogenesis Recovery Potentials after Transplantation of Adipose Tissue-Derived Mesenchymal Stem Cells Cultured with Growth Factors in Experimental Azoospermic Mouse Models

**DOI:** 10.22074/cellj.2020.6055

**Published:** 2019-07-31

**Authors:** Masoumeh Eliyasi Dashtaki, Masoud Hemadi, Ghasem Saki, Javad Mohammadiasl, Ali Khodadadi

**Affiliations:** 1 Cellular and Molecular Research Center, School of Medicine, Ahvaz Jundishapur University of Medical Sciences, Ahvaz, Iran; 2 Physiology Research Center, School of Medicine, Ahvaz Jundishapur University of Medical Sciences, Ahvaz, Iran; 3 Department of Medical Genetics, School of Medicine, Ahvaz University of Medical Sciences, Ahvaz, Iran; 4 Cancer, Environmental and Petroleum Pollutants Research Center, Ahvaz Jundishapur University of Medical Sciences, Ahvaz, Iran

**Keywords:** Azoospermia, Epidermal Growth Factor, Glial Cell Line-Derived Neurotrophic Factor, Leukemia Inhibitory Factor, Mesenchymal Stem Cells

## Abstract

**Objective:**

Approximately 1% of the male population suffers from obstructive or non-obstructive azoospermia. Previous *in vitro* studies have successfully differentiated mesenchymal stem cells (MSCs) into germ cells. Because of immune- modulating features, safety, and simple isolation, adipose tissue-derived MSCs (AT-MSCs) are good candidates for such studies. However, low availability is the main limitation in using these cells. Different growth factors have been investigated to overcome this issue. In the present study, we aimed to comparatively assess the performance of AT-MSCs cultured under the presence or absence of three different growth factors, epidermal growth factor (EGF), leukemia inhibitory factor (LIF) and glial cell line-derived neurotrophic factor (GDNF), following transplantation in testicular torsion-detorsion mice

**Materials and Methods:**

This was an experimental study in which AT-MSCs were first isolated from male Naval Medical Research Institute (NMRI) mice. Then, the mice underwent testicular torsion-detorsion surgery and received bromodeoxyuridine (BrdU)-labeled AT-MSCs into the lumen of seminiferous tubules. The transplanted cells had been cultured in different conditioned media, containing the three growth factors and without them. The expression of germ cell-specific markers was evaluated with real-time polymerase chain reaction (PCR) and western-blot. Moreover, immunohistochemical staining was used to trace the labeled cells.

**Results:**

The number of transplanted AT-MSCs resided in the basement membrane of seminiferous tubules significantly increased after 8 weeks. The expression levels of Gcnf and Mvh genes in the transplanted testicles by AT-MSCs cultured in the growth factors-supplemented medium was greater than those in the control group (P<0.001 and P<0.05, respectively). The expression levels of the c-Kit and Scp3 genes did not significantly differ from the control group.

**Conclusion:**

Our findings showed that the use of EGF, LIF and GDNF to culture AT-MSCs can be very helpful in terms of MSC survival and localization.

## Introduction

Infertility is among the common health issues worldwide that affects 15% of couples. Among infertile men, approximately 1% of the cases suffers from obstructive or non-obstructive azoospermia, with the latter being difficult to treat (1). One of the causes involved in the non-obstructive azoospermia is testicular torsion (2). Recently, researchers have offered a new approach to the treatment of infertility that involves differentiating stem cells into male or female germ cells **in vitro** (3-6). Adipose tissue- derived mesenchymal stem cells (AT-MSCs) have high proliferation rate and self-renewal capacity, as well as the potential to differentiate into various lineages (7). Recent studies have shown that both embryonic and adult stem cells are able to differentiate into primordial germ cells (PGCs) and adult gametes (4, 8, 9). In 2006, Nayernia et al. (4, 9) demonstrated the production of a generation of mice from germ cells derived from embryonic stem cells (ESCs) for the first time, and in the same year, they were able to differentiate murine bone marrow-derived MSCs (BM-MSCs) into germ cells. Zhang et al. (10) recently reported that BM- MSCs have the potential to trans-differentiate into sperm-like cells, and can revive fertility in busulfantreated azoospermic rats. Similarly, Cakici et al. (11) have shown that AT-MSCs cause regeneration of fertility in azoospermic rats. 

However, amongst the important issues for therapeutic applications of these produced cells are their low numbers and viability (12). To overcome these problems, growth factors and several supplements are often added to the culture media of these cells (13-15). Epidermal growth factor (EGF) is a 53 amino acid protein (16) involved in proliferation of spermatogonia and regulation of spermatogenesis in mammalian testis (17). It is also involved in the proliferation of MSCs (14). Leukemia inhibitory factor (LIF) is involved in the self-renewal process of stem cells, maintenance of the non-differentiated forms of ESCs, MSCs, and proliferation of PGCs (18, 19). The glial cell line- derived neurotrophic factor (GDNF) is expressed by glial cells in the brain (20), testicular and ovarian tissues during the development, and it has been found to be responsible for spermatogonial stem cells (SSCs) self-renewal both *in vitro* and *in vivo* (21). The present study is aimed to compare the performances of AT- MSCs cultured with or without the addition of three different growth factors EGF, LIF, and GDNF to their culture medium, following their transplantation in testicular torsion-detorsion mice. 

## Materials and Methods

### Animals 

In this experimental study, 6-8 week-old male Naval Medical Research Institute (NMRI) mice were housed under standard conditions (18-20°C and 12:12 hours light: dark cycles) at the Research Center and Experimental Animal House of Jundishapur University of Medical Sciences (Ahvaz, Iran). All the experiments presented in this study were approved by The Local Animal Care Committees of Ahvaz Jundishapur University of Medical Sciences (AJUMS) (IR.AJUMS.REC.2015.739), which were in complete accordance with the guidelines for the care and use of laboratory animals set by the national academy of sciences (National Institutes of Health Publication No. 86-23). 

### Isolation and culture of adipose tissue derived mesenchymal stem cells

Adipose tissue was taken from epididymis of 5-10 male NMRI mice in a sterile environment. Then, the samples were washed three times with phosphatebuffered saline (PBS, Gibco Life Technologies, Paisley, UK) containing 3% penicillin/streptomycin(Pen/Strept) and 0.3% amphotericin B, then cut into1-2 mm^3^ pieces. Blood vessels were removed fromthe tissue as much as possible and the pieces of fat were incubated in collagenase type I enzyme (Sigma- Aldrich, St. Louis, MO, 1 mg/ml) for 25-30 minutes at 37°C. To stop the enzyme activity, Dulbecco’s Modified Eagle’s Medium (DMEM, Life Technologies, USA) containing 10% fetal bovine serum (FBS, Gibco, Life Technologies, USA) was added to the sample. The suspension sample was centrifuged at 1200 rpm for 7 minutes, at room temperature and the cell pellet was cultured in 25 cm^2^ flasks containing DMEM medium supplemented with 15% FBS and 1% Pen/Strep, and incubated at 37ºC in the presence of 5 % CO^2^. After three days, the cell medium was replaced with fresh medium and non-cohesive cells were removed. Medium was changed once every two days until the cell density reached 80-90%. The cells were then passaged for more proliferation and purification. For this purpose, 1 ml 0.25% trypsin-ethylenediaminetetraacetic acid (EDTA) was added to each flask and incubated for 2-3 minutes. When the cells were floating in the flask, the trypsin was neutralized using 3-4 ml of the medium containing FBS. Then, the cell suspension was centrifuged for 7 minutes. at 1200 rpm and the cell pellet was cultured in new flasks at a density of 20000/cm^2^ (22) . 

### Adipose tissue-derived mesenchymal stem cells identification 

MSCs are fibroblast analogue cells with adhesion property and differentiation capacity (23). However, before any transplantation it is necessary to confirm the exact type of the cells isolated from donor animals. For this purpose, we used the commonly applied flow cytometry technique to confirm specific cell surface markers on the cultured cells. The expression of CD90 and CD44 markers (specific to MSCs) and the lack of expression of the two CD31 and CD45 markers (specific to hematopoietic stem cells and endothelial cells) were investigated, in our previous study (22).

### *In vitro* osteogenic and adipogenic differentiation potentials of adipose tissue-derived mesenchymal stem cells 

In order to further characterize of our cultured cells, we assessed their ability to differentiate into osteoblasts and adipocytes. For osteogenic differentiation, AT-MSCs (passage 3) were cultured in a 6-well plate (5×10<sup>4</sup> cells/well). After 24 hours, the proliferative medium was replaced with osteogenic differentiation medium [DMEM (low glucose), 10^-7^ M dexamethasone, 50 µg/ml ascorbic acid , and 10 mM B-glycerol phosphate (Sigma-Aldrich, St. Louis, Mo, USA)]. The cells were incubated at 37°C and in 5% CO2 for 21 days. Osteogenic medium was exchanged every 3 days. At the end of the differentiation period, AT-MSCs were fixed with 3% paraformaldehyde and the presence of calcium deposits was examined using 0.5 % alizarin red solution. 

For adipogenic differentiation, 5×10^4^ cells/well (passage 3) were seeded in a 6-well plate. After 24 hours, the proliferative medium was replaced with adipogenic differentiation medium [DMEM (low glucose) supplemented with 10^-7^ M dexamethasone, 66 nM insulin, 0.2 mM indomethacin and 0.5 mM isobutylmethylxanthine (Sigma-Aldrich, St. Louis, Mo, USA)]. The cells were incubated at 37°C and 5% CO2. After 14 days, the cells were fixed with 3% paraformaldehyde and the presence of lipid follicles was examined by Oil Red O staining (0.5% in methanol, Sigma-Aldrich, St. Louis, Mo, USA) (22, 24). 

### Bromodeoxyuridine labeling of adipose tissue-derived mesenchymal stem cells

For labeling the AT-MSCs prior to transplantation we used Bromodeoxyuridine (BrdU), which is a base analogue substituted for thymine during DNAsynthesis in proliferating cells. Following the denaturation of double-stranded DNA, BrdU is detected by immunohistochemistry, thus a population of cells that has proliferated is identified (25). Using this method we were able to trace the transplanted cells in the murine testicles. To do so, passage 3 AT-MSCs were incubated in 10 mM BrdU (Sigma-Aldrich, St. Louis, Mo, USA) overnight and BrdU immunohistochemistry kit (Merck, Germany) was used to confirm labeling of the cells. 

### Induction of azoospermia by surgical testicular torsion-detorsion procedure 

To create azoospermic mice, we used the testicular torsion-detorsion method. For this purpose, twenty 6-8 week-old male NMRI mice (25-30 g) were first anesthetized by intraperitoneal injection of ketamine and xylazine, then the scrotal midline was cut, tunica vaginalis was opened and the testicle was twisted 720 degrees around its axis in a counterclockwise direction and was fixed with a 4-0 silk suture. Two hours later (26), the testicle was untwisted and fixed to the scrotal wall, which was then surgically closed (27). The right testicle of each group was also considered as the positive control for that group. 

### Labeled adipose tissue-derived mesenchymal stem cells transplantation

Six weeks after testicular torsion-detorsion surgery, the mice were anesthetized with ketamine/xylazine, scrotal walls were opened and 10^5^ AT-MSCs were injected into the lumen of seminiferous tubules of testicular torsion-detorsion mice by Hamilton syringes. Testicles were fixed in their places and scrotal walls were closed again. The mice were divided into three groups. Group 1 was injected with AT-MSCs cultured in EGF (10 ng/ml), LIF (5 ng/ml), and GDNF (5 ng/ ml) (MSCs-GF group), group 2 was injected with AT- MSCs that were cultured in a medium without growth factors (MSCs-T group), and group 3 was the testicular torsion-detorsion mice that did not receive any cells (negative control). The right testicles of all mice were considered as the positive control group for each treatment. To verify that the injected AT-MSCs have entered the testicles, the cells were stained with trypan blue. 8 weeks after cell transplantation, 5 testicles in each group were removed for molecular analysis. For histological analyses, 3 testicles in each group were removed and fixed in formalin and hematoxylin-eosin staining and immunohistochemical analysis were performed on tissue sections. 

### Hematoxylin-eosin staining

For histological assessment, hematoxylin-eosin staining was done. The stages of staining were performed according to the standard protocols, as summarized in the study by Cardiff et al. (28). All reagents were from Sigma-Aldrich (St. Louis, Mo, USA). 

### Immunohistochemical assessments of testicles

In order to trace the AT-MSCs labeled with BrdU, immunohistochemical staining was performed. For this purpose, the tissues containing BrdU were fixed in 4% formalin, then dehydrated and embedded in paraffin. Five-micron thick slices were prepared from the paraffin blocks and placed on slides for immunostaining. The slides were kept at 37°C overnight. Prior to staining, the sections were deparaffinized, then staining was performed according to the BrdU immunohistochemistry kit (Merck, Germany) instructions. 

### RNA extraction, cDNA synthesis and real time polymerase chain reaction

The RNeasy Mini Kit (Qiagen, Germany) was used to extract the total tissue RNA as per the company instructions. The cDNA was synthesized with Quanti Nova Reverse Transcription Kit (Qiagen, Germany) according to the company instructions. Primers for the selected genes were designed specifically using Gene Bank sequences. Primer sequences of *c-Kit, Mvh, Scp3, Gcnf* and *Gapdh* are as follows respectively:

c-Kit- F: 5´-GAGAAGGAAGCGTGACTCGT-3´
  R: 5´-TCTTGCGGATCTCCTCTTGT-3´,
Mvh- F: 5´-CGAAACATAGGTGATGAAAGAAC-3´
  R: 5´-CCACTGAAGTAGCAACAAGAAC-3´,
Scp3 F: 5´-AAAGCATTCTGGGAAATCTG-3´
  R: 5´-GTACTTCACCTCCAACATCTTC-3´,
Gcnf- F: 5´-CAACTGAACAAGCGGTATT-3´
  R: 5´-GATGTATCGGATCTCTGGC-3´,
Gapdh- F: 5´-AAGGTCATCCCAGAGCTGAA-3´
  R: 5´-CTGCTTCACCACCTTCTTGA-3´.

Quantitative real-time polymerase chain reaction (qRT-PCR) stages were performed in Applied Biosystems 7500 Sequence Biosystem. Briefly, 100 nM of the primers and 100 ng cDNA were added to Syber Green PCR master mix to reach the overall volume of 10 µl, then the reaction was carried out in 45 cycles, at 95°C for 15 seconds and 58-60°C for 1 minutes. The gene expression levels in every sample were normalized with the *Gapdh* gene and data was evaluated using 2^-ΔΔCT^ approach (22). 

### Western blot analysis

To assess the expression of c-Kit and Gcnf proteins, western blot analysis was performed. In this method, tissue samples were lysed in radioimmunoprecipitation assay buffer (RIPA) solution [150 mM NaCl, 25 mM Tris-HCl (pH=7.6), 1% Triton X-100, and, 1 mM EDTA pH=7.4, 3% sodium dodecyl sulfate (SDS, Sigma- Aldrich, St. Louis, Mo, USA), 1% Sodium deoxy collate] supplemented with 0.1% phosphatase inhibitor (Sigma-Aldrich, USA). The concentrations of the proteins was specified using bicinchoninic acid assay (BCA assay). The equivalent quantity of the protein samples (60 µg) was loaded on 12% polyacrylamide gel, and then transferred to polyvinylidene fluoride (PVDF) membrane (Amersham, UK). PVDF membrane was blocked at room temperature for one hour in Tris Buffer/Tween 20 (TBST) solution containing 3% skim milk. Then, the membrane was incubated with primary antibodies in the blocking buffer at 4°C overnight: Gcnf (1:1000, Abcam, USA), c-Kit (1:250, Abcam, USA), ß-actin (1:250, Santa Cruz Biotechnology, Germany). After washing with TBST, the membrane was exposed to the secondary antibody in the blocking buffer [goat anti rabbit IgG-HRP (1:15000, Abcam, USA)] for one hour at room temperature. The membrane was then washed in TBST and enhanced chemiluminescence (ECL) western blotting substrate (Abcam, USA) was used for detection of the protein bands according to the manufacturer’s instructions. Beta-actin protein was used as a loading control. Image J software was used to measure and compare the density of the protein bands in the experimental and control groups. 

### Statistical analysis

For the analysis of the real time PCR tests, the relative expression levels of the genes were calculated by the 2-..CT formula and SPSS version 16 (SPSS Ink,. USA) was used for statistical analysis. All quantitative variables were expressed as mean ± SD. The variations were evaluated using one way analysis of variance (ANOVA), Kruskal- Wallis test, Dunnett test and LSD test. For all statistical analyses, the statistical significance was set as P=0.05.

## Results

### Isolated adipose tissue-derived mesenchymal stem cells characterization 

**Fig.1:**
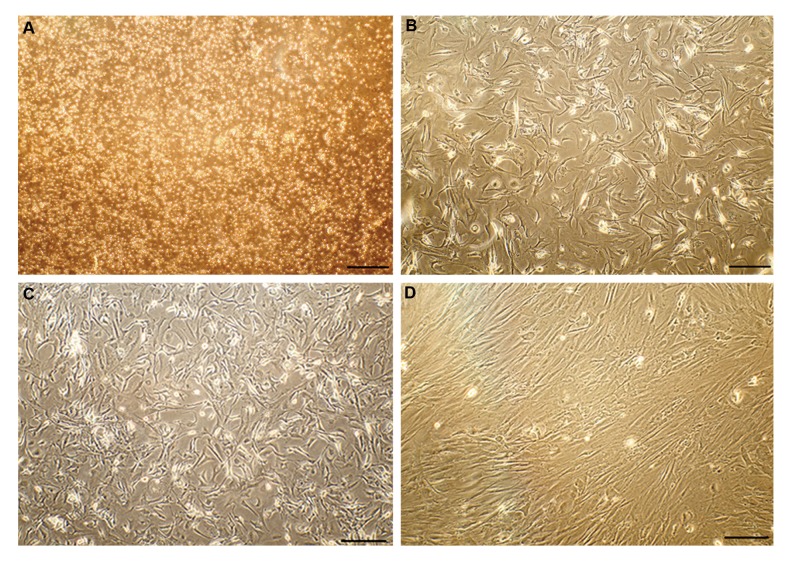
Morphology of the cultured adipose tissue derived mesenchymal stem cells. **A.** Day 0, **B.** Day 3, **C.** Day 5, and **D.** Passage 1 (scale bar: 100 µm).

AT-MSCs were isolated from the adipose tissue around epididymis of male NMRI mice as was explained before. On the first day, the isolated cells were round shaped, but three days later, the cells became spindle-shaped and fibroblast-like. Other types of cells including endothelial and blood cells were also seen in the flask, however, these cells were eliminated during passaging (Fig .1). 

**Fig.2:**
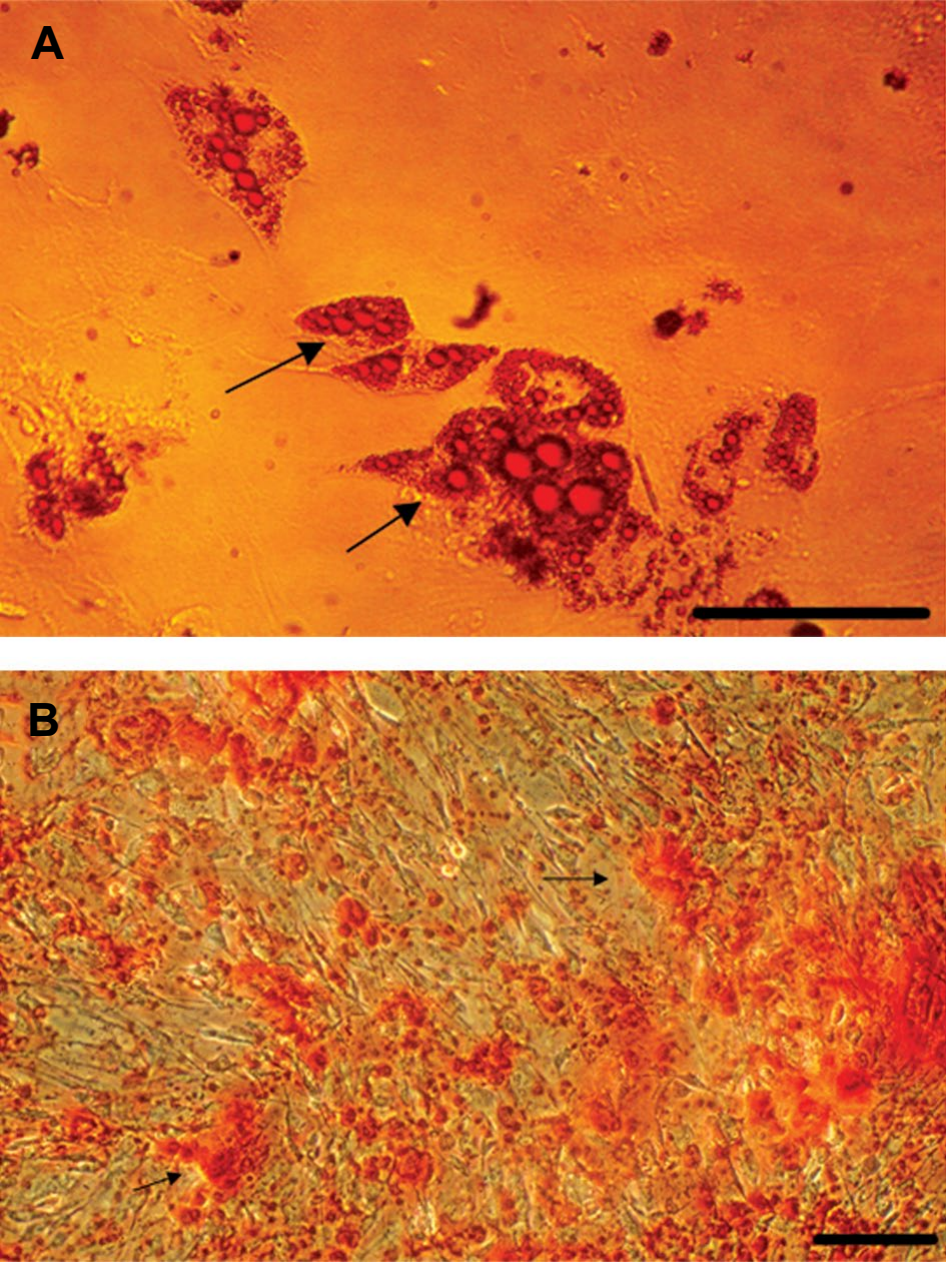
Adipose tissue derived mesenchymal stem cells exhibited stem cell characteristics. **A.** Adipocyte differentiation of the cells. Arrows showlipid vacuoles stained with oil red O and **B.** Differentiation of the cells into osteocytes. The arrows show the calcium nodules stained with alizarin red(scale bar: 100 µm).

Nine days after the induction of adipogenic differentiation, lipid vacuoles within the cells were observed. After 14 days, the cells were stained with Oil Red O and the lipid particles turned red (Fig .2A). The first signs of change in the morphology of AT-MSCs and differentiation to osteocyte cells were seen 10 days after inducing osteogenic differentiation. After 21 days, the cells started forming calcium nodules. The cells formed mineral matrixes around themselves that were visible by Alizarin Red staining (Fig .2B). The results showed that these cells have the potentials to differentiate into both adipogenic and osteogenic lineages.

According to the results of our previous study, the isolatedcells expressed high levels of CD90 and CD44 markers, andshowed low expressions of CD31 and CD45. These values indicated a high level of purity of the isolated MSCs (22). 

### Histological analysis of recipient mice testis

**Fig.3:**
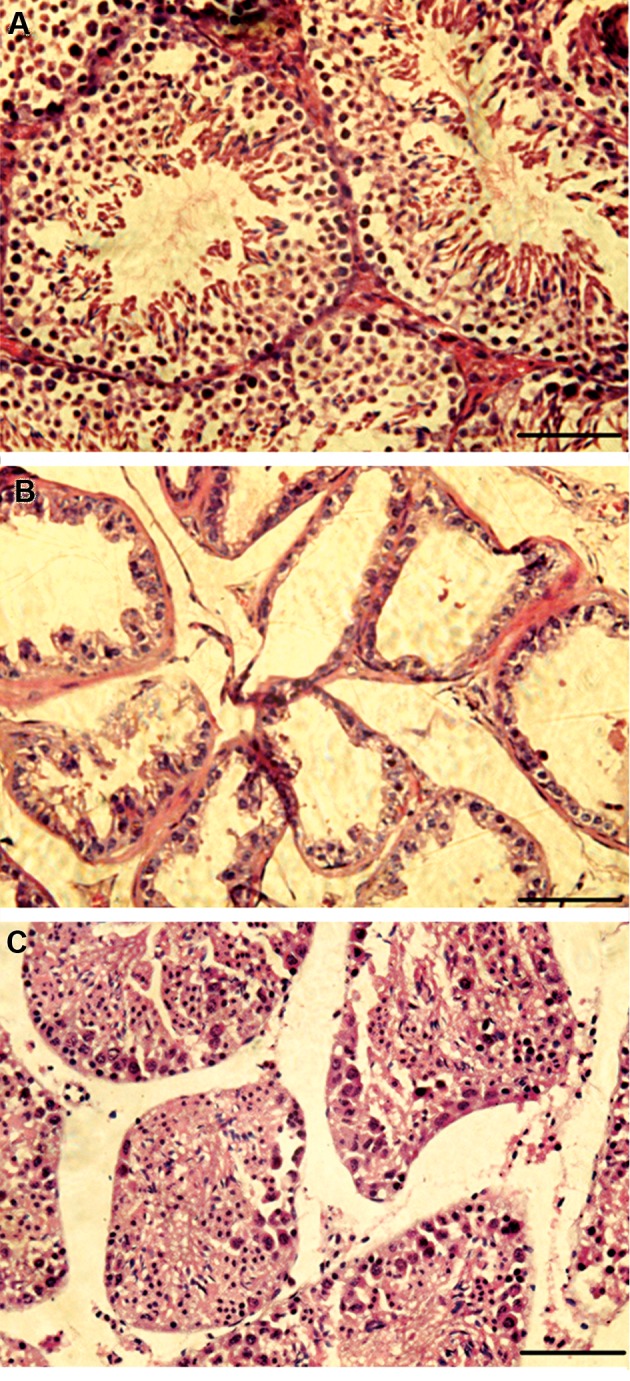
H&E staining of testis sections. **A.** Positive control (scale bar: 50 µm), **B.** Torsion testis: six weeks after the torsion/detorsion, most of sperm cells were eliminated, and **C.** Cell transplanted testis after 8 weeks, spermatogenesis was observed in seminiferous tubules (scale bar: 100 µm).

**Fig.4:**
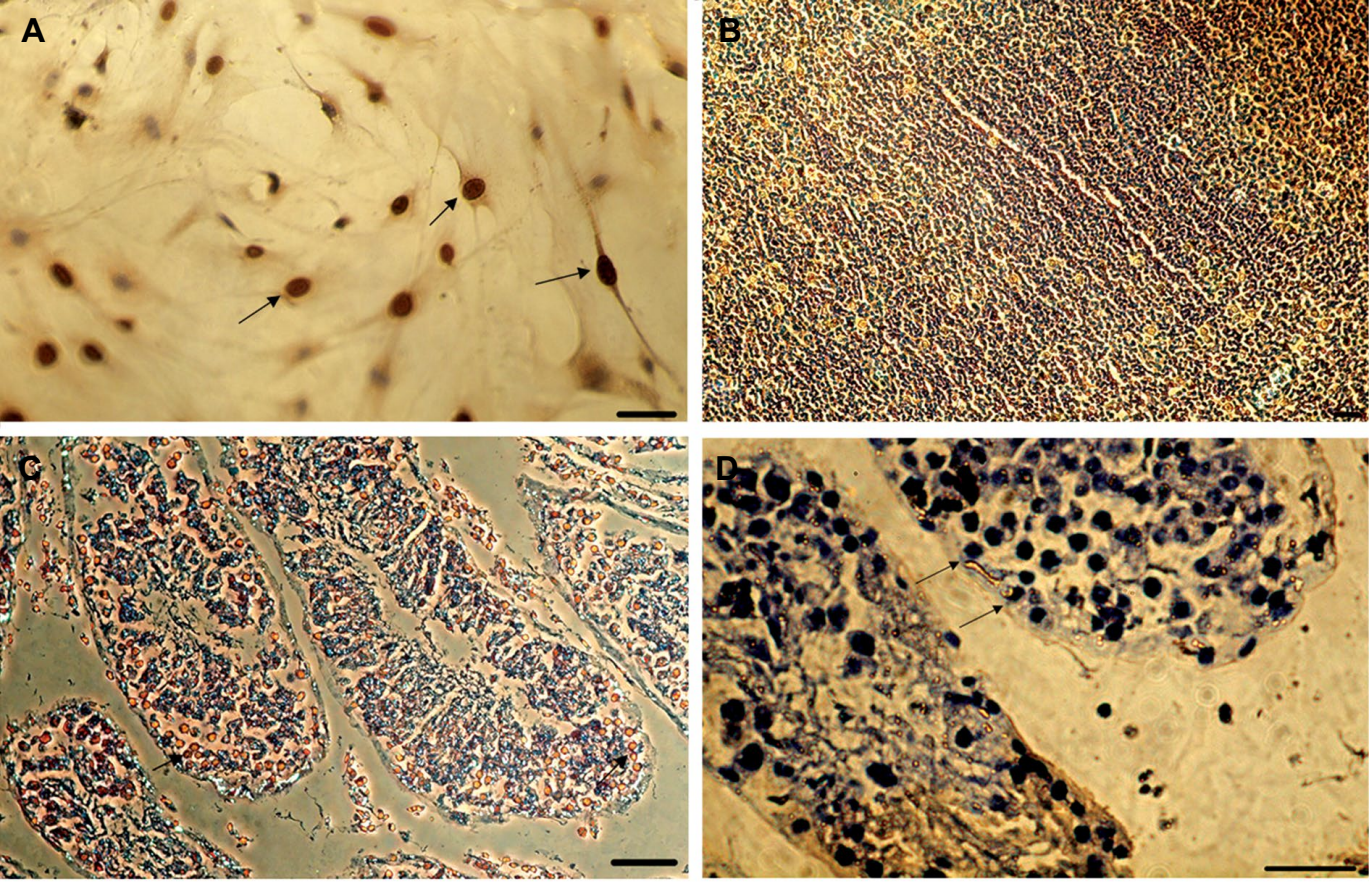
Bromodeoxyuridine (BrdU) staining of the cells and tissues. **A.** The labeled cells with BrdU before transplantation. Arrows show the labeled cells, **B.** Positive control, intestinal mouse cells, **C,** and **D.** BrdU labeled cells transplanted into mouse testis. Most of the cells were localized into the basement membrane of seminiferous tubules (the brown cells in C and the dark cells in D). BrdU-labeled cells are shown by arrows (scale bar: 50 µm).

Six weeks after torsion-detorsion surgery of mice, hematoxylin-eosin staining of testicle tissue sections was performed. In the seminiferous tubules of the testicular torsion-detorsion mice, most of the sperm cells were eliminated, spermatogenesis was arrested and the tubules were empty from spermatogenic cells, while Sertoli cells and seminiferous tubules structures were maintained (Fig .3). Eight weeks after cell transplantation, most of the labeled cells had survived and were resided in the basement membrane of the seminiferous tubules. Spermatogenesis process successfully occurred in seminiferous tubules and spermatogenic cells were observed in these tubules (Fig .4).

### Expression of spermatogenic molecular markers in testicle of transplanted mice 

**Fig.5:**
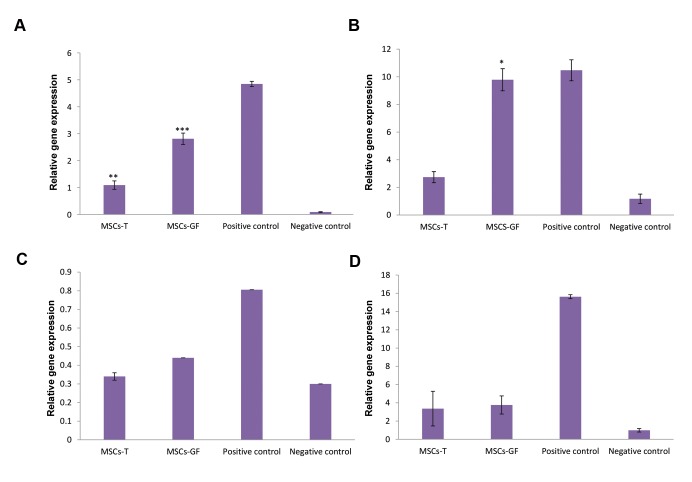
Expression of spermatogenic molecular markers in testicle of the transplanted mice. **A.** The *Gcnf* expression in MSCs-GF group (P<0.001) and MSCs-T group (P<0.01) increased compared to the control group, **B.** The expression of *Mvh*, in the MSCs-GF group showed a significantly higher level than the control group (P<0.05), **C,** and **D.** Expression of *Scp3* and *c-Kit* markers showed no significant difference compared to the control group. *; P<0.05, **; P<0.01, ***; P<0.001, MSCs; Mesenchymal stem cells, AT-MSCs; Adipose tissue-derived MSCs, MSCs-T; The group of mice injected with AT-MSCs, and MSCs-GF; The group of mice injected with AT-MSCs cultured with growth factors, torsion: negative control.

The expression levels of *Gcnf* gene, a germ cell-specificmarker, in both MSCs-GF (P<0.001) and MSCs-T groups(P<0.01) increased significantly compared to the control group. *Gcnf* gene expression in the MSCs-GF group wassignificantly higher than that in MSCs-T group (P<0.001). The expression level of *Mvh* another germ cell-specificmarker, in the MSCs-GF group was significantly highercompared to the control group (P<0.05). The expression ofthis gene was not significantly different in the MSCs-T group. The expression levels of *Scp3* and *c-Kit* markers showed no significant difference in either experimental group compared to the control group (Fig .5). 

### Protein analysis after adipose tissue-derived mesenchymal stem cell transplantation 

**Fig.6:**
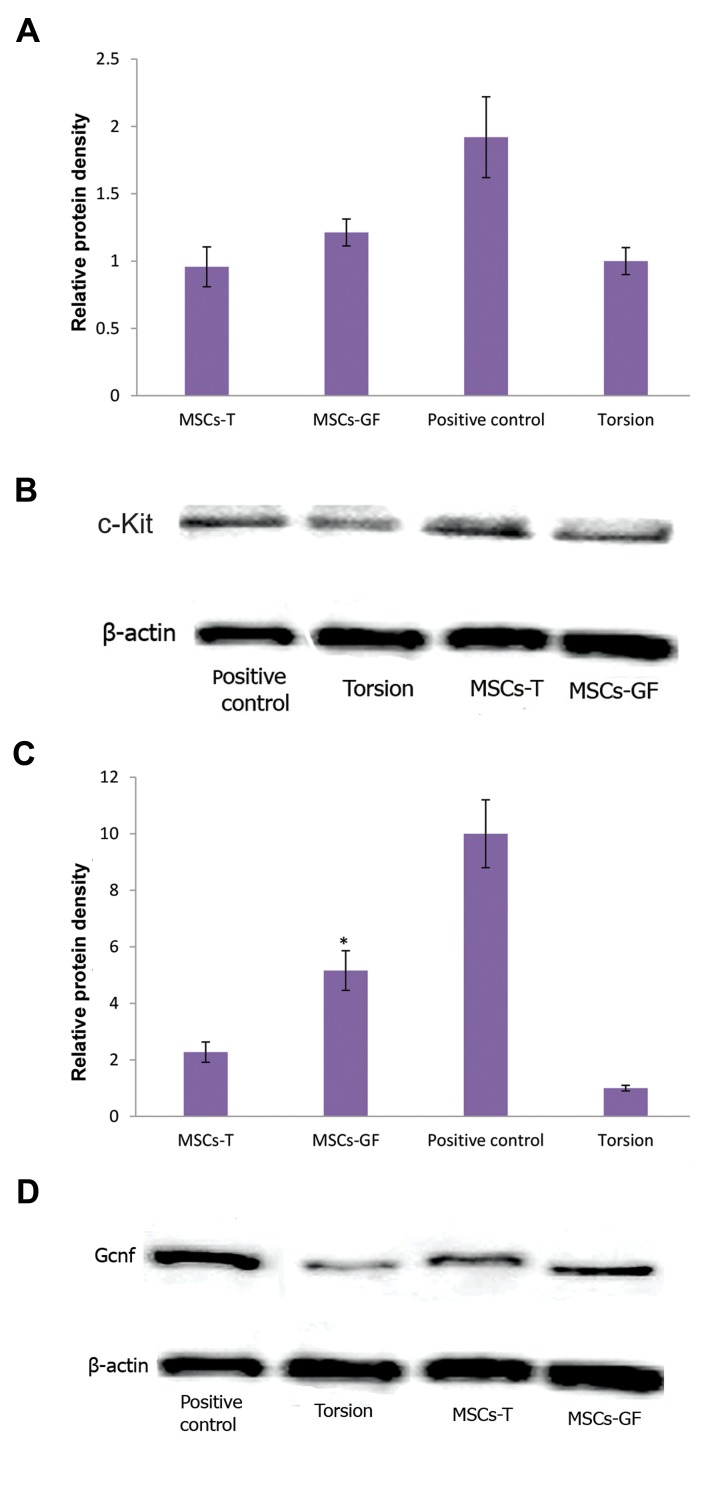
Western blot results. **A, B.** c-Kit protein expression results (not statistically significant), **C,** and **D.** Expression of Gcnf protein in the MSCs-GF group was higher but the MSCs-T group showed no significant difference compared to the control group. *; P<0.05, MSCs; Mesenchymal stem cells, AT-MSCs; Adipose tissue-derived MSCs, MSCs-T; The group of mice that received AT-MSCs, and MSCs-GF; The group of mice that received AT-MSCs cultured with growth factors, torsion: negative control.

c-Kit protein expression in both MSCs-GF and MSCs-T groups showed no difference compared to the control groups and confirmed the results of the real time PCR method. Expression of Gcnf protein in the MSCs- GF group was higher than the control group (P<0.05), but the MSCs-T group showed no significant difference compared to the control group (Fig .6). 

## Discussion

Stem cell-based therapy has become one of the new potential treatment for the near future in regenerative medicine for the repair of damaged tissues and organs in many diseases such as infertility (29). AT-MSCs can be obtained easily and are highly capable of proliferation and differentiation into different lineages. These fibroblast- like cells have high immune-modulating properties. Therefore, they are considered appropriate options for autologous cell transplantation (30).

Most of the previous studies show that MSCs confer potential of spermatogenesis recovery in azoospermic animal models (5, 31-33). Nayernia et al. (9) was the first group who reported that murine (BM)-MSCs possess a high differentiating potential into male germ cells. Zhang et al. (10) demonstrated that BM-MSCs have the capability of differentiating into sperm-like cells and restoring fertility in busulfan treated azoospermic rats Ghasemzadeh- Hasankolaei’s group (34) as well as Anand et al. (35) have also reported similar results. Vahdati et al. (32) have shown that BM-MSCs revive spermatogenesis of infertile hamsters. Consistent with our results, Cakici et al. (11) also showed the restoration of the fertility of azoospermic rats after injection of AT-MSCs. Hsiao et al. (33) have reported that through inhibition of apoptosis and enhancement of testosterone secretion, MSCs prevent infertility in torsion rats. These findings indicate that MSCs successfully differentiate into germ cells in animal models and have the potentials to be used in the treatment of infertility in human patients as well. 

In the present study, one group of mice was injected with AT-MSCs cultured in a medium supplemented with EGF, LIF, and GDNF growth factors. These factors increase proliferation and viability of the AT-MSCs *in vitro* (22). They are also secreted in the testicular niche that influences the proliferation process and maintenance of SSCs (17, 21). Spermatogenesis process occurs in seminiferous tubules. The core components of testicular niche include the basement membrane, Sertoli cells, peritubular myoid cells, and the extracellular signaling molecules. Sertoli cells are one of the most important of these components and provide the necessary growth factors for proliferation and maintenance of SSCs (36). We injected the cells into the semniferous tubules of azoospermic mice. In fact, an appropriate microenvironment was provided for differentiation of MSCs. In previous studies, a busulfantreated azoospermic mouse model was used as recipient (10, 11). One limitation of using this model is damaging seminiferous tubules structure and destroying the testicular niche, especially Sertoli cells through busulfan treatment. 

Transplanted AT-MSCs migrate to the basement membrane of seminiferous tubules. They are affected by the seminiferous tubules niche and factors secreted by Sertoli cells, and begin to differentiate. In studies conducted by Zhang et al. (10) and Cakici et al. (11), a small number of the cells remained in the basement membrane after injection. However, in our study, after 8 weeks, a large number of AT-MSCs were resided in the seminiferous tubules basement membrane, which can be due to the increase of viability of the cells induced by the growth factors as well as an appropriate mouse model, in which the structure of seminiferous tubules and testicular niche have been maintained.

After 8 weeks, *Gcnf* gene expression in the cell- transplanted groups (MSCs-GF and MSCs-T) was significantly higher compared to the control group. Interestingly, MSCs-GF group showed further increased expression of the germ cell-specific markers (*Mvh, Gcnf*). It could be due to the impact of the growth factors on viability of the cells. In addition, EGF, LIF, and GDNF are secreted from sertoli cells and testicular niches (17, 21). Therefore, they are probably effective in the process of differentiation of the injected cells into germ cells. Expression of *c-Kit* and *Scp3* genes in the AT-MSC recipient groups did not significantly differ from the control group. These genes are related to the final stages of sperm differentiation (37). However, this could be due to our short tracking time. Zhang et al. (10) showed increased expression of *Mvh* and *Gcnf* factors in the testicular tissues and reported that *c-Kit* expression was reduced after 8 weeks. The exact mechanism of function of the transplanted MSCs has not been specified. There are several possibilities in this regard: i. MSCs are differentiated into target tissue cells under the influence of their niche (38), ii. MSCs secrete factors, which stimulate inner stem cells or lead to revival of damaged tissue (7), and iii. In the case of infertility, MSCs can prevent infertility by inhibition of oxidative stress and apoptosis (33).

The observations in the present study may indicate that the injected AT-MSCs have entered spermatogenic pathway or have revived damaged testicular tissue and SSCs by secretion of trophic factors. To determine its exact mechanism, the cells should be tracked over a longer period of time in the future studies.

## Conclusion

This study showed that the transplanted AT-MSCs were localized in the basement membrane of seminiferous tubules. The testicles of the mice injected with AT-MSCs expressed spermatogenesis-specific markers. The mice that received cells that were cultured in the presence of growth factors showed overexpression of germ cell- specific markers. According to these results, the use of EGF, LIF and, GDNF to culture AT-MSCs can be very helpful in terms of MSC survival and localization, but further preclinical studies in different animal models and with different time points are needed to develop an effective clinical application. 
